# Quality of life following cardiac rehabilitation in cardiac surgery patients

**DOI:** 10.1186/s13019-022-01893-9

**Published:** 2022-05-31

**Authors:** Ernest Christian Lourens, Robert Ashley Baker, Bronwyn M. Krieg

**Affiliations:** CTSU Quality and Outcomes, Flinders Medical Centre, Bedford Park, Adelaide, SA 5042 Australia

**Keywords:** Cardiac surgery, Coronary artery bypass graft, CABG, Health-related quality of life, HRQOL, Cardiac rehabilitation, Barriers to uptake

## Abstract

**Background:**

Undergoing cardiac surgery often result in perioperative loss of health-related quality of life (HRQOL). Although participation rates in Australia is low, cardiac rehabilitation (CR) has been demonstrated to improve patient HRQOL in cardiac patients. Literature is unclear regarding the role of CR and HRQOL in the cardiac surgery (CS) patient population.

**Methods:**

A prospective non-randomised study was conducted on eligible cardiac surgery patients between December 2009 and March 2015. HRQOL was assessed using the Short Form 12 at baseline and post-operatively at 30 days and 180 days. CR participation was recorded and barriers to CR uptake was assessed using the Cardiac Rehabilitation Enrolment Obstacles (CREO) scale.

**Results:**

At 180 days, 107 patients participated in CR and 111 did not participate in CR. A significant improvement from baseline mental and physical HRQOL was observed in both groups at 30 days and 180 days (*p* < 0.002). No significant difference between group characteristics or HRQOL was observed at any time. A trend of superior improvement in mental QOL was observed in the CR group. The study is limited by poor initial uptake (218/1772 of eligible) and may be underpowered to observe a clinical difference. A significant difference in CREO scores were observed between the two groups at 30 days (13 out of 16 questions, *p* < 0.001) and 180 days (11 out of 16 questions, *p* < 0.011).

**Conclusion:**

Literature has shown that CR may improve numerous health outcomes in cardiac and CS patients, however CR uptake in Australia is low. Mental and Physical QOL is demonstrated to improve following CS, however further research is required to delineate the role of CR and QOL in CS patients. The CREO tool utilised in this study identified numerous potentially modifiable barriers to CR uptake. Specific strategies related to the survey are suggested to improve awareness, uptake, and adherence to CR, including advocacy of home-based and telehealth services.

## Introduction

Cardiovascular disease is the largest cause of mortality in the Australian population, with disease sequelae sometimes requiring surgical intervention [1]. Undergoing cardiac surgery (CS) such as heart valve replacement or coronary artery bypass grafting (CABG) has a significant impact on patients’ physical and emotional health. Following disease sequelae and surgical convalescence, patients frequently report a loss of health-related quality of life (HRQOL) and physical functioning before and after surgery [2]. It is therefore important to study postoperative strategies that may improve health outcomes.

Cardiac rehabilitation (CR) is a multi-disciplinary intervention provided to patients following acute coronary syndrome (ACS) or cardiac surgery [3]. Programs typically involve medical evaluation, risk-factor modification, education, prescriptive exercise, counselling and behavioural interventions. The goal of CR is to provide optimal physical and mental conditions, attenuate or reverse disease progression and prevent disability through improved health behaviour [2–4].

Recent studies in CS patients have demonstrated that CR is associated with improved exercise capacity and reduced, all-cause mortality and overall morbidity [2, 4–8]. CR has proven so effective that it has a Level 1A recommendation on the National Health and Medical Research Council level of evidence following ACS [9]. HRQOL following CS is reported in the literature, however the effect of CR is unclear [1, 2]. Anderson et al. [4] investigated CR for coronary heart disease and found in 14 out of 20 of included studies reported an increased HRQOL in one or more HRQOL subscales in CR groups compared with controls, however a meta-analysis could not be undertaken due to heterogeneity of data. In 2009, Lie et al. [9] demonstrated in a randomised control trial a significant increase in HRQOL following CAG in both the control and CR groups, however they reported no meaningful difference between the two groups. Similarly, in a randomised control trial study involving heart valve surgery (n = 147) an improvement in exercise capacity following CR was demonstrated, but no difference in HRQOL (SF36) compared to controls [10]. A prospective cohort study conducted in Washington (n = 947) found no meaningful improvement in HRQOL due to CR after CABG [11]. More research is therefore required to delineate the role of CR in HRQOL following CS.

Despite the beneficial evidence of CR, uptake and adherence is sub optimal, in Australia CR referral rates have been reported to as low as 46%, with available CR spaces vastly underutilised [3, 12]. At Flinders Medical Centre (FMC) the CR participation rate for the period December 2009 to March 2015 was 28%, identifying a clear need for intervention. There are many barriers to uptake and participation in CR described in literature, some of which will be discussed in this study.

This study aimed to quantify changes in HRQOL following CS in the presence or absence of CR, as well as to identify barriers to CR uptake using the Cardiac Rehabilitation Enrolment Obstacles (CREO) scale.

## Materials and methods

### Study design

This was a prospective non-randomised study of CS patients at FMC from December 2009 to March 2015. All patients undergoing CABG, mitral valve replacement (MVR), aortic valve replacement (AVR) and transcatheter aortic valve implantation (TAVI) were eligible for inclusion. The study aimed to include all patients willing to consent, no power calculations were performed. Participants were required to be more than 18 years old, English speaking and be willing and able to sign the informed consent form. Emergency CS procedures were excluded. Patients were able to withdraw from the study at any time.

### Ethics approval and consent to participate

Ethics approval was granted by the South Adelaide Health Service / Flinders University, Flinders Clinical Research Ethics Committee, Flinders Medical Centre (FWA0001785) on 5 November 2009. Flinders Medical Centre, Level 6, Ward 6C, Room 6A219, Flinders Drive, Bedford Park, SA 5042. Telephone: + 61 8 8204 6061.

### Participant assessment

Upon enrolment of participants into the study, a baseline evaluation was completed to determine HRQOL and functional status prior to surgery. Follow-up assessments were completed at 30 days (30d) and 180 days (180d) postoperatively. Patients medical records were reviewed to determine mortality and hospital re-admission status and were contacted via telephone to complete study questionnaires. Data on patient demographics, clinical outcomes and CR program screening and participation was collected in the FMC Cardiac Surgery Registry. Participants underwent routine clinical management in all cases.

### Study questionnaires

HRQOL was assessed using the Short Form 12 (SF12) (“Appendix 1”) [14] which has been previously employed in the cardiac patient population [15] and covers domains endorsed by the World Health Organisation as a requisite for a HRQOL measure [16]. Functional status was determined using the New York Heart Association (NYHA) classification [17]. Perceived barriers to CR participation was assessed using the Cardiac Rehabilitation Enrolment Obstacles (CREO) scale [18].

### Cardiac rehabilitation program

All patients who underwent CS were invited to attend CR. This study’s CR program was based upon a previously established model [3]. There was no additional cardiac rehabilitation offered to patients who participated in this study. Patients were provided with a Heart Foundation booklet and consulted by a CR team consisting of dieticians, pharmacists, social workers, physiotherapists and CR nurses [19]. Educational sessions (60 min duration) and the group-based exercises circuits (30–60 min) were conducted twice a week for 6 weeks.

### Statistical analysis

Health outcomes were entered in the software program IBM SPSS (version 21.0) for statistical analyses. Comparison of between-group difference were done using an independent samples t-test. Normal distribution of data was assessed using the Shapiro–Wilk and Kolmogorov–Smirnov tests prior to computation of results. Analysis of study outcomes were analysed using a mixed-model analysis of variance for repeated measures. A level of significance of *p* = 0.05 was used and Bonferroni-corrected level of significance was applied. Any participant surveys that were incomplete or contained missing data were not included in the analysis. Differences in categorical group characteristics were tested using the Fisher’s exact test.

## Results

### Participants

**Fig. 1:**
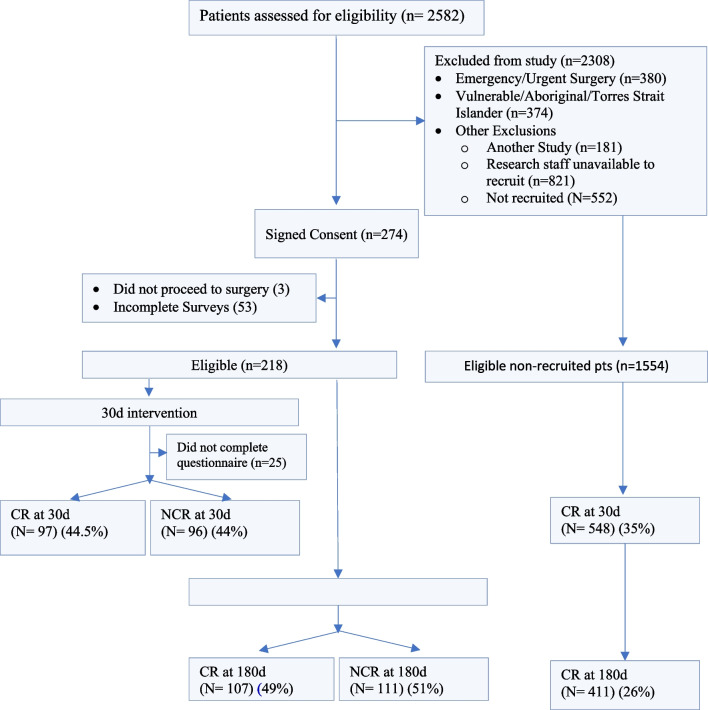
Consort diagram characteristics of participating patients. The consort diagram illustrates of 2582 patients eligible for Cardiac Surgery (CS), 218 completed sufficient questionnaires. Of those completing questionnaires, 44.5% attended Cardiac Rehab (CR) at 30 days, 11% did not complete a questionnaire and 44% did not complete CR (NCR). At 180 days 49% attended CR, and 51% did not. Of eligible patients who did not complete questionnaires, 35% completed CR at 30 days and 26% at 180 days, identifying a need to address barriers to CR uptake and adherence over 180 days

From 15 December 2009 to 16 March 2015, 2582 patients underwent CS at FMC. Of these patients, 218 met the inclusion criteria, agreed to participate in the study and completed the questionnaires (Fig. 1). No statistical differences in baseline characteristics, age, sex, comorbidities, New York Heart Association Scale (NYHA) class or procedure type were observed between the CR and Non-Cardiac Rehabilitation (NCR) groups (Table 1).

**Table 1:** Baseline characteristics of participating patients

	CR	NCR	Sig. (*p* =)
*Patient demographics*			
Number of participants: (M/F)	107 (69/41)	111 (65/46)	0.526
Age years: mean (SD)	69.01 (11.1)	68.62 (11.0)	0.799
BMI (SD)	29.83 (6.15)	30.10 (6.5)	0.753
Past medical history of:			
T2 diabetes (n)	82	85	0.992
Hypertension	88	93	0.957
CHF	60	60	0.764
Smoking	91	100	0.258
NYHA class (SD)	1.97 (0.9)	1.93 (0.9)	0.743
*Procedure type*^a^			
CAG	44	46	0.962
Aortic valve	56	57	0.883
Mitral valve	17	20	0.675
Percutaneous/TAVI	5	6	0.805
Hospital readmission (all cause)	27	26	0.755

CR = cardiac rehabilitation, NCR = non-cardiac rehabilitation, M/F = male/female, SD = standard deviation (n) = number, CHF = congestive heart failure, NYHA = New York Heart Association Scale (Appendix 3)

^a^NB some patients underwent more than one procedure type

### SF12 health related quality of life (HRQOL)

There was no significant difference in physical HRQOL between CR and NCR groups at baseline (*p* = 0.476), 30d (*p* = 0.830) and 180d (*p* = 0.617) (Table 2). There was also no significant difference in mental HRQOL between CR and NCR groups at baseline (*p* = 0.229), 30d (*p* = 0.114) and 180d (*p* = 0.591).

**Table 2:** CR and NCR physical and mental QOL

CR Group	N =	Mean	SD	Sig. from CR baseline	Sig. from CR 30d	Sig. from NCR
SF12 physical QOL						
Baseline	97	37.51	11.21	–	–	*p* = 0.476
30d	97	41.97	8.75	*p* = 0.002	–	*p* = 0.830
180d	107	47.36	10.00	*p* = 0.000	*p* = 0.000	*p* = 0.617
SF12 mental QOL						
Baseline	97	45.97	12.29	–	–	*p* = 0.229
30d	97	53.03	10.86	*p* = 0.000	–	*p* = 0.114
180d	107	53.91	9.32	*p* = 0.000	*p* = 0.548	*p* = 0.591

NCR Group	N =	Mean	SD	Sig. from NCR baseline	Sig. from NCR 30d	Sig. from CR
SF12 physical QOL						
Baseline	96	36.42	9.93	–	–	*p* = 0.476
30d	96	41.71	8.04	*p* = 0.000	–	*p* = 0.830
180d	111	46.67	10.31	*p* = 0.000	*p* = 0.000	*p* = 0.617
SF12 mental QOL						
Baseline	96	47.98	10.74	–	–	*p* = 0.229
30d	96	55.21	7.99	*p* = 0.000	–	*p* = 0.114
180d	111	54.59	9.32	*p* = 0.000	*p* = 0.672	*p* = 0.591

A statistically significant difference in mental and physical HRQOL was observed from baseline to 30d and baseline to 180d in CR and NCR groups. A statistically significant improvement from 30 to 180d in physical QOL was also observed for both CR (*p* = 0.000) and NCR groups (*p* = 0.000). A similar trend was not observed in for mental HRQOL. No group showed a relative superior improvement from baseline to 30d or 180d in either physical or mental domains. The NCR group demonstrated an insignificant decline in Mental HRQOL from 30 to 180d (m = 55.21, SD = 7.99 to m = 54.59, SD = 9.32) whereas the CR group continued to demonstrate an insignificant increase from 30 to 180d (m = 53.03, SD = 10.86 to m = 53.91, SD = 9.32).

### Barriers to participation

**Fig. 2:**
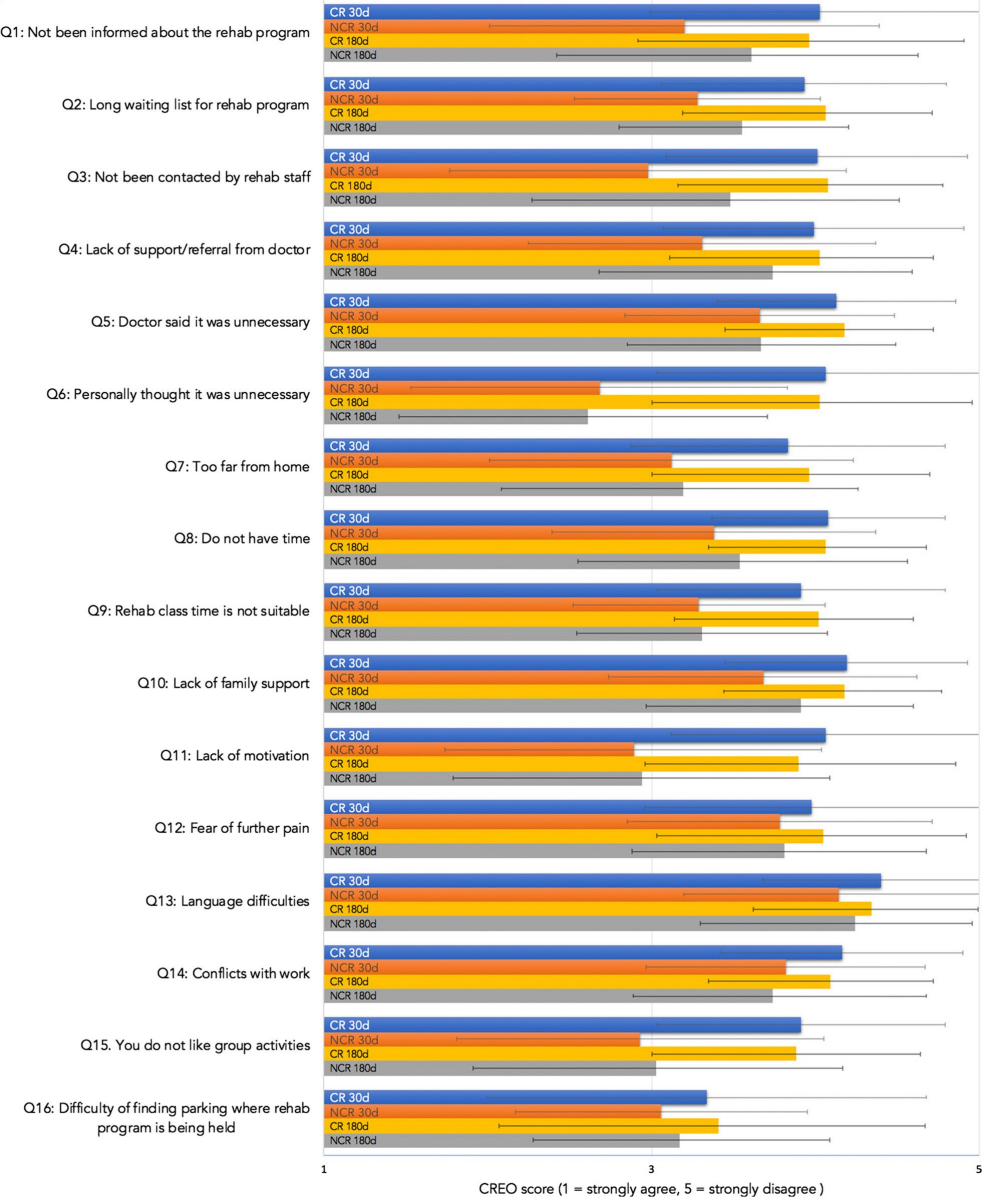
CREO scores per question for CR and NCR groups at 30d and 180d. Visually demonstrates the data from Table 2; changes in CREO scores from 30 days (30d) to 180 days (180d) in the Cardiac Rehabilitation (CR) and Non-Cardiac Rehabilitation (NCR) groups. A significant difference between CR and NCR groups can be observed in Q1–11 and Q14–15 at 30d and Q2–3, Q5–11 and Q14–15 at 180d

Of all study participants, 44.5% attended CR at 30d and 49% by 180d, compared to 35% of non-recruited patients who attended CR at 30 days and 26% at 180 days (Fig. 1). The Cardiac Rehabilitation Enrolment Obstacles (CREO) survey highlighted some of the barriers to the uptake of CR after CS (Fig. 2, “Appendix 2”). A total of 172 (CR = 87) participants provided responses at 30d and 211 (CR = 103) at 180d. A significant difference was found between CR and NCR group responses in 13 out of 16 questions at 30 days and 11 out of 16 at 180 days (Table 3, Fig. 2). The questions showing the largest variance between the groups included patients responding that they have not been contacted by staff (Q3), personal belief that CR was unnecessary (Q6), live too far from nearest CR centres (Q7), are time poor (Q8), lack motivation (Q11) or dislike group activities (Q15). At 30d and 180d participants in the NCR group also believed the waiting list was too long (Q2), their doctor said it was unnecessary (Q5) and that the class schedules were not suitable (Q9 and Q14). Participants in the NCR group were also more likely to report a lack of family support regarding CR (Q10).

**Table 3:** CREO scores for CR and NCR at 30d and 180d

	CR (n = 87)		NCR (n = 85)		Sig
	Mean	SD	Mean	SD	
**30d**					
*Question*					
Q1: Not been informed about the rehab program	4.03	1.04	3.20	1.19	***p* < 0.001**
Q2: Long waiting list for rehab program	3.93	0.87	3.28	0.75	***p* < 0.001**
Q3: Not been contacted by rehab staff	4.01	0.92	2.98	1.21	***p* < 0.001**
Q4: Lack of support/referral from doctor	3.99	0.92	3.31	1.06	***p* < 0.001**
Q5: Doctor said it was unnecessary	4.13	0.73	3.66	0.82	***p* < 0.001**
Q6: Personally thought it was unnecessary	4.06	1.03	2.68	1.15	***p* < 0.001**
Q7: Too far from home	3.83	0.96	3.12	1.11	***p* < 0.001**
Q8: Do not have time	4.08	0.71	3.38	0.99	***p* < 0.001**
Q9: Rehab class time is not suitable	3.91	0.88	3.29	0.77	***p* < 0.001**
Q10: Lack of family support	4.19	0.74	3.68	0.94	***p* < 0.001**
Q11: Lack of motivation	4.06	0.94	2.89	1.15	***p* < 0.001**
Q12: Fear of further pain	3.98	1.02	3.78	0.93	*p* = 1.000
Q13: Language difficulties	4.40	0.72	4.14	0.94	*p* = 0.147
Q14: Conflict with work	4.16	0.74	3.82	0.85	***p* = 0.047**
Q15: You do not like group activities	3.91	0.88	2.93	1.12	***p* < 0.001**
Q16: Difficulty of finding parking	3.34	1.34	3.06	0.89	*p* = 0.558
Total	63.27	9.57	53.20	6.44	***p* < 0.001**

	CR (n = 103)		NCR (n = 108)		
	Mean	SD	Mean	SD	
**180d**					
*Question*					
Q1: Not been informed about the rehab program	3.96	0.95	3.61	1.02	*p* = 0.095
Q2: Long waiting list for rehab program	4.06	0.65	3.55	0.65	***p* < 0.001**
Q3: Not been contacted by rehab staff	4.08	0.70	3.48	1.03	***p* < 0.001**
Q4: Lack of support/referral from doctor	4.03	0.69	3.74	0.85	*p* = 0.101
Q5: Doctor said it was unnecessary	4.18	0.54	3.67	0.82	***p* < 0.001**
Q6: Personally thought it was unnecessary	4.03	0.93	2.61	1.10	***p* < 0.001**
Q7: Too far from home	3.96	0.74	3.19	1.07	***p* < 0.001**
Q8: Do not have time	4.06	0.62	3.54	1.02	***p* < 0.001**
Q9: Rehab class time is not suitable	4.02	0.58	3.31	0.76	***p* < 0.001**
Q10: Lack of family support	4.18	0.59	3.91	0.69	***p* = 0.042**
Q11: Lack of motivation	3.90	0.96	2.94	1.15	***p* < 0.001**
Q12: Fear of further pain	4.05	0.87	3.81	0.87	*p* = 0.390
Q13: Language difficulties	4.34	0.65	4.24	0.72	*p* = 1.000
Q14: Conflict with work	4.09	0.63	3.74	0.94	***p* = 0.011**
Q15: You do not like group activities	3.88	0.76	3.03	1.14	***p* < 0.001**
Q16: Difficulty of finding parking	3.41	1.26	3.17	0.92	*p* = 0.701
Total	64.23	5.99	55.54	6.35	***p* < 0.001**

Bold indicates *p* < 0.05

## Discussion

### Study outcomes

This study demonstrates a significant improvement in physical and mental HRQOL following CS in the CR and NCR groups at 30d and 180d, consistent with current literature demonstrating that CS results in HRQOL improvements [2, 4]. A significant improvement in physical HRQOL was observed from 30 to 180d in both groups suggesting ongoing improvement in QOL following cardiac surgery. However, no statistical differences were observed between the CR and NCR groups at baseline, 30d or 180d in physical and mental HRQOL categories. These results are consistent with a previous literature review demonstrating that HRQOL improves following CS but with inconclusive evidence that CR following CS improves HRQOL superiorly to control groups [4, 9–11].

Barriers to participating in CR were identified using the CREO, with most questions demonstrating a statistical difference between the CR and NCR group at 30d and at 180d. The validity of CREO as a CR scale has previously been established to have good internal consistency (Cronbach's alpha = 0.89) and divergent validity [18]. Results from this study are similar to Fernandez (2008), demonstrating a significantly poorer CREO scores for CS patients who choose not to participate in CR [18]. The CREO tool provides a helpful way of determining barriers to CR, to date however its report throughout literature is limited [19]. These identified barriers form a basis for intervention to improve future CR uptake and adherence at FMC and other centres.

### Srategies to conquer barriers and increase CR uptake

By analysis the results from the CREO data, it is clear that early uptake of CR is largely dependent on participants’ active awareness of the program (Q1), being contacted by CR staff (Q3) and having an early referral and support from their doctor (Q4, Q5). It is therefore a recommended that a streamlined process is put in place where all patients are educated about CR as early as possible during the patient journey, including having immediate access to online, home-based CR resources and telehealth access. By allowing participants to have a bridging online access to CR telehealth education and exercise classes, it reduces the immediate problem of long CR waiting lists (Q2), travel distance from home (Q7), class times beings unsuitable (Q8,Q9), potential conflicts with work schedule (Q14), dislike of group activities (Q15), driving/parking difficulties (Q16) as well as to combat Coronavirus (COVID-19) population health, travel, social distancing and household isolation restrictions [21]. Previous studies have indicated that home-based exercises programmes have higher completion rates and similar efficacy to centre-based classes, therefore providing all patients with home exercise education and regular phone follow-up may reduce CR barriers to uptake and adherence [22, 23]. Similarly, providing patients with written information packs and pre-recorded educational videos and home exercise instructions may increase exercise adherence and familial support (Q10-11). Dedicated goal-setting, action planning, self-monitoring and regular feedback has also demonstrated to increase CR uptake and adherence [12]. Studies have also shown increased compliance and outcomes when CR participants receive 3–5 text messages per week containing health and lifestyle modification advice [24]. The use of mobile tracking applications and wearable devices to encourage and track physical activity is becoming more readily available and recognised in literature, with recent studies demonstrating up to 87% use adherence study participants [25]. Such devices can aid participants in monitoring their own physical activity in a way which is meaningful to each participant, therefore increasing the likelihood of ongoing adherence (Q6,11). Other strategies described in literature include; automated referrals of all eligible patients, CR referral included in all discharge plans, third-party (e.g. ward-clerk) redundancy check of referral, written invitation provided to patient, comprehensive use of interpreter service, arrangement of transport if required and persistent follow-up of non-attendees [26]. Another barrier to CR is patient belief that CR is unnecessary and lack of motivation (Q6 and 11). It is therefore recommended that CR include directed patient-centric education and goal setting upon diagnosis, that relays the benefits of CR following CS and thereby improve patient belief regarding CR importance and to incite motivation. Finally, recent studies suggest that rehabilitation before surgery (termed prehabilitation) may have numerous improvements in health outcomes including HRQOL and post-operative pulmonary complications [27–30], therefore overall patient health-related outcomes, CR uptake and adherence may be increased if CR is commenced prior to CS.

### Limitations

This study had a number of limitations. Of all potentially eligible patients, 12% (218/1772) agreed to participate in the study, while 60% were not approached due to limited research staff availability. The poor participation and requirement capacity exposes this study to potential selection bias. No power calculation was performed prior to study commencement. The study was designed as a non-randomised, prospective cohort study. Participants were given the choice of CR participation, therefore some patients participated only at 30 or 180 days (not both) and therefore was not included in the final analysis (Fig. 1). Similarly, 13% of the participants did not complete a questionnaire at 30 days, limiting result reliability. Participants who did not complete a questionnaire at 180d were not included in the analysis. It is therefore likely that the sample size was too small to detect group differences in HRQOL at the study endpoints. The study design lacks a reproducible exercise regime, and program adherence was self-reported and not quantified. Future studies may utilise an evidence-based and reproducible CR exercise program as described in literature [26]. This study used the SF12 to assess HRQOL which may not be sensitive enough to detect differences in HRQOL experienced by patients undergoing CR or not. A potentially more sensitive alternative may include the Short Form 36 (SF36), EuroQol EQ-5D or similar [4]. In the context of the current global environment, future studies may benefit from investigating the effect of CR on HRQOL using telehealth, online-resources, home-based CR and self-monitoring using mobile applications and wearable devices.

## Conclusion

This study found that patients who undergo cardiac surgery have significant improvement in mental and physical quality of life at 30 days and again at 180 days. Approximately half of the recruited patients participated in cardiac rehabilitation. There was no difference in HRQOL between participants who participated in CR and those who did not. The study is limited by poor initial uptake (218/1772 of eligible) and may be underpowered to observe a clinical difference. Using the CREO tool, a significant difference in responses was found between the CR and NCR group in 13 out of 16 questions at 30 days, identifying numerous potentially modifiable barriers to CR uptake. Specific strategies related to the survey are suggested to improve awareness, uptake, and adherence to CR.

## Data Availability

The datasets used and/or analysed during the current study are available from the corresponding author on reasonable request.

## Appendices

### Appendix 2: Cardiac Rehabilitation Enrolment Obstacles (CREO)

**Table:**

Number	Question	Response: (1–5)*
1	Not been informed about the rehab program	
2	Long waiting list for rehab program	
3	Not been contacted by rehab staff	
4	Lack of support/referral from doctor	
5	Doctor said it was unnecessary	
6	Personally thought it was unnecessary	
7	Too far from home	
8	Do not have time	
9	Rehab class time is not suitable	
10	Lack of family support	
11	Lack of motivation	
12	Fear of further pain	
13	Language difficulties	
14	Conflict with work	
15	You do not like group activities	
16	Difficulty of finding parking where rehab program is being held	

*Response options

1 = Strongly agree

2 = Agree

3 = Unsure

4 = Disagree

5 = Strongly disagree

### Appendix 3: New York Heart Association (NYHA) classification

**Table:**

Class	Definition
I	No limitations. Ordinary physical activity does not cause undue fatigue, dyspnoea or palpitations (asymptomatic LV dysfunction)
II	Slight limitation of physical activity. Ordinary physical activity results in fatigue, palpitation, dyspnoea or angina pectoris (mild CHF)
III	Marked limitation of physical activity. Less than ordinary physical activity leads to symptoms (moderate CHF)
IV	Unable to carry on any physical activity without discomfort. Symptoms of CHF present at rest (severe CHF)

## Abbreviations

ACS: Acute coronary syndrome
AVR: Aortic valve replacement
CABG: Coronary artery bypass graft
CR: Cardiac rehabilitation
CREO: Cardiac rehabilitation enrolment obstacles
CS: Cardiac Surgery
FMC: Flinders Medical Centre
HRQOL: Health-related quality of life
MVR: Mitral valve replacement
NCR: Non-cardiac rehabilitation
NYHA: New York Heart Association
QOL: Quality of life
SD: Standard deviation
TAVI: Transcatheter aortic valve implantation
